# Aquatic Thermal Reservoirs of Microbial Life in a Remote and Extreme High Andean Hydrothermal System

**DOI:** 10.3390/microorganisms8020208

**Published:** 2020-02-03

**Authors:** Vilma Pérez, Johanna Cortés, Francisca Marchant, Cristina Dorador, Verónica Molina, Marcela Cornejo-D’Ottone, Klaudia Hernández, Wade Jeffrey, Sergio Barahona, Martha B. Hengst

**Affiliations:** 1 Laboratorio de Ecologia Molecular y Microbiologia Aplicada, Departamento de Ciencias Farmacéuticas, Facultad de Ciencias, Universidad Católica del Norte, Antofagasta 1240000, Chile; vilma.perez@adelaide.edu.au (V.P.); j.cortes.guzman021@gmail.com; 2 Australian Centre for Ancient DNA (ACAD), University of Adelaide, Adelaide, SA 5005, Australia; 3 Centro de Biotecnología y Bioingeniería (CeBiB), Universidad de Chile, Santiago 8320000, Chile; f_marchant_m@hotmail.com (F.M.); cristina.dorador@uantof.cl (C.D.); 4 Laboratorio de Complejidad Microbiana y Ecología Funcional, Instituto Antofagasta & Departamento de Biotecnología, Facultad de Ciencias del Mar y Recursos Biológicos, Universidad de Antofagasta, Antofagasta 1240000, Chile; barahona.sergio.l@gmail.com; 5 Observatorio de Ecología Microbiana, Departamento de Biología Facultad de Ciencias Naturales y Exactas, Universidad de Playa Ancha, Valparaíso 2340000, Chile; veronica.molina@upla.cl; 6 Escuela de Ciencias del Mar & Instituto Milenio de Oceanografía, Pontificia Universidad Católica de Valparaíso, Valparaíso 2340000, Chile; marcela.cornejo@pucv.cl; 7 Centro de Investigación Marina Quintay CIMARQ, Facultad de Ecología y Recursos Naturales, Universidad Andrés Bello, Santiago 8320000, Chile; claudia.hernandez.r@unab.cl; 8 Center for Environmental Diagnostics & Bioremediation, University of West Florida, Pensacola, FL 32514, USA; wjeffrey@uwf.edu; 9 Laboratorio de Microbiología Aplicada y Extremófilos, Universidad Católica del Norte, Antofagasta 1240000, Chile

**Keywords:** NGS, 16S rRNA, microbial diversity, archaea, thermophiles, terrestrial hydrothermal systems, Lirima

## Abstract

Hydrothermal systems are ideal to understand how microbial communities cope with challenging conditions. Lirima, our study site, is a polyextreme, high-altitude, hydrothermal ecosystem located in the Chilean Andean highlands. Herein, we analyze the benthic communities of three nearby springs in a gradient of temperature (42–72 °C represented by stations P42, P53, and P72) and pH, and we characterize their microbial diversity by using bacteria 16S rRNA (V4) gene metabarcoding and 16S rRNA gene clone libraries (bacteria and archaea). Bacterial clone libraries of P42 and P53 springs showed that the community composition was mainly represented by phototrophic bacteria (Chlorobia, 3%, Cyanobacteria 3%, at P42; Chlorobia 5%, and Chloroflexi 5% at P53), Firmicutes (32% at P42 and 43% at P53) and Gammaproteobacteria (13% at P42 and 29% at P53). Furthermore, bacterial communities that were analyzed by 16S rRNA gene metabarcoding were characterized by an overall predominance of Chloroflexi in springs with lower temperatures (33% at P42), followed by Firmicutes in hotter springs (50% at P72). The archaeal diversity of P42 and P53 were represented by taxa belonging to Crenarchaeota, Diapherotrites, Nanoarchaeota, Hadesarchaeota, Thaumarchaeota, and Euryarchaeota. The microbial diversity of the Lirima hydrothermal system is represented by groups from deep branches of the tree of life, suggesting this ecosystem as a reservoir of primitive life and a key system to study the processes that shaped the evolution of the biosphere.

## 1. Introduction

Early Earth was characterized by high temperatures in primitive oceans, high UV radiation, low oxygen concentrations, acidic pH, intensive volcanic activity, and a reduced atmosphere with high hydrogen concentrations [1,2,3]. One of the theories about the origin of primary life proposes that it may have occurred in thermal environments such as deep-sea hydrothermal vents [4,5], and the common ancestor of life (LUCA) was probably a chemolithoautotrophic, thermophilic, and anaerobic organism [6,7]. Therefore, the study of modern hydrothermal ecosystems could provide a better comprehension about the evolution of earliest life in the primitive atmosphere [8]. 

High altitude ecosystems around the Atacama Desert contain several hot springs that are characterized by extreme environmental factors such as those encountered in early earth, presenting extreme aridity, high UV radiation, and a wide daily temperature oscillation. The geochemical composition of the water has been reported to be dominated by sodium sulfate, chloride, and arsenic [9]. These unique environmental and geochemical conditions create peculiar niches for microbial life that are potentially similar to early Earth. In fact, hot springs have been reported to be microbial-dominated ecosystems, supporting a rich microbial diversity and containing several microhabitats that constitute natural niches for thermophilic (>50 °C) and hyperthermophilic (>80 °C) microorganisms, such as archaea and bacteria (and their viruses) [10,11,12]. High-altitude hydrothermal systems represent extremely fragile ecosystems that are often exploited for groundwater uptake and geothermal energy generation in industrial processes such as the mining industry without considering the uniqueness of these environments and the ecosystem services that provides for indigenous communities living here. 

The remote Lirima hydrothermal system is located in the north of Chile, 25 km from the Sillaiguay volcanic chain at 4000 m a.s.l., close to the Aroma-Quimchasata volcanic complex (19°51′24″ S, 68°55′02″ W), one of the most tectonically active areas in the world with extensive geothermal and volcanic activity [9,13,14,15]. This environment is surrounded by a mountain desert, and it has been recognized as a complex polyextreme environment for life [16,17,18,19,20]. Microbial communities from Lirima hot springs have not been comprehensively studied. To our knowledge, there is only a single microbial diversity survey based on a culture-dependent approach that identified five bacterial phyla belonging to Gammaproteobacteria, Alphaproteobacteria, Firmicutes, Actinobacteria and Bacteroidetes [21]. Considering the above, we characterize the microbial diversity of three closely located hot springs in the Lirima hydrothermal system by using both 16S rRNA gene clonal (bacteria and archaea) and 16S rRNA (V4 region) gene metabarcoding analyses, and we determine the influence of physical and chemical environmental conditions on those microbial community structures. The results obtained in this study are discussed in the context of how hydrothermal systems are important reservoirs of unexplored microbial diversity and crucial systems to understand essential questions concerning the metabolisms, adaptations, and phylogenetic relationships of these microbial communities with deep branched lineages in the tree of life.

## 2. Materials and Methods

### 2.1. Description of the Sampling Sites

**Figure 1:**
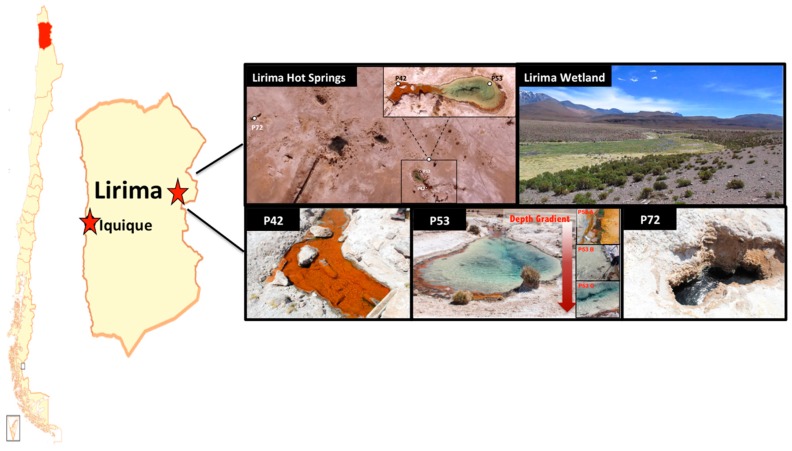
Lirima hydrothermal system. Map of Chile that displays the location of the Lirima hydrothermal system and the spatial distribution of the hot springs (P42, P53A, P53B, P53C, and P72).

Samples for physical, chemical and biological analyses were collected from three permanent pools at the hydrothermal Lirima ecosystem (i.e., P42, P53 (A, B and C), and P72, which were named according to the water temperature) at the end of the dry season and the beginning of the wet season (during April 2011 for the 16S rRNA gene clone libraries and November 2013 for the 16S rRNA gene metabarcoding analysis) (Figure 1; Table 1). P42 is a shallow pool (40 cm depth) that is colonized at the bottom by a thick and compact orange mat. P53 is a deeper (1.5 m) transparent water pool with orange mats growing only at the surface of the external margin. The sediment below the mat is a thin greyish layer. Three sediment samples were analyzed from this pool: P53A (surface, mat), P53B (medium, sediment), P53C (bottom, sediment). P72 is the hottest pool, with dark greyish sediments and a depth of 1.6 m.

### 2.2. Sample Collection and Physical–Chemical Analysis

Mat and sediment samples were collected in sterile 50 mL Falcon tubes from each pool. Samples for enrichment cultures were stored at 4 °C upon collection until cultured in the laboratory. Samples for molecular analysis were immediately preserved in RNALater (Ambion, Life Technologies, Austin, TX, USA) and stored to 4 °C upon collection until they were processed in the laboratory. Physical and chemical parameters were obtained in situ, with a multiparameter instrument (Hanna HI991301) measuring temperature, pH, conductivity, turbidity, and redox potential (Table 1). Additional discrete water samples were taken during November 2011 for CO_2_, CH_4_ and N_2_O gas analyses (Table S1) and during November 2013 for the chemical characterization of 37 parameters including alkalinity, nutrients (total nitrogen, nitrite, nitrate, sulfate, phosphate, and silicate) and metals, all of which were analyzed by using standard methods (Table S1). The samples for gas determination were taken in 20 mL vials and preserved with 50 µL of saturated HgCl_2_ to inhibit biological activity, and the samples were then stored in the dark until analysis by the headspace technique of McAullife [22]. The analysis was conducted in a gas chromatograph that was equipped with an electron capture detector (ECD) for N_2_O determination, a methanizer to convert CO_2_ to CH_4_, and an flame ionization detector (FID) to determine CH_4_ concentration (GC Greenhouse 2014, Shimadzu, Kyoto, Japan).

### 2.3. Enrichment Cultures and Scanning Electron Microscopy (SEM)

Environmental samples were collected during April 2011. Enrichment cultures of microbial mats and sediment samples were performed to increase the number of cells to detectable levels to determine the presence of microbial communities and their morphologies. For enrichment cultures, one gram of each sample was inoculated into 10 ml of Medio Salar 1% NaCl (0.5 g L^−1^ casein peptone, 0.2 g L^−1^ yeast extract, 0.1g L^−1^ NaCl). The enrichment cultures were incubated for 21 days, without shaking, at specific temperatures that corresponded to those of the sampling sites under aerobic conditions in the dark. The growth cultures were prepared for SEM by dehydration in a graded ethanol series (10–100%) followed by critical point drying. The samples were analyzed with a Scanning Hitachi 3000 SEM (Hitachi, Japan) at the Pontificia Universidad Católica de Chile.

### 2.4. Clonal Analysis of Archaeal and Bacterial 16S rRNA Gene

Environmental samples were collected during April 2011. The mat samples from each site were shaken vigorously and homogenized prior to DNA extraction. For DNA extractions, 0.25 g of homogenized samples were used. All DNA extractions were carried out by using the UltraClean Soil DNA isolation kit (MoBio Laboratories Inc., Carlsbad, CA, USA) according to the manufacturer’s instructions. DNA quality and concentration were evaluated by 1% *w/v* agarose gel electrophoresis and with a Nanodrop spectrophotometer (Nanodrop 2000c, Thermo Scientific, Waltham, MA, USA). 

The amplification of the archaeal 16S rRNA gene was performed by using a nested PCR approach based on that of Jurgens et al. [23] with the primers Ar4F and Un1492R (~1500 bp), and then a second PCR was performed by using this amplicon as template but with the primers Ar3F and Ar9R (~900 bp). Each PCR reaction contained 1× PCR buffer with 3 mM MgCl2 (Promega), 400 µM of deoxynucleotide triphosphates (dNTP) mixture (Promega, Madison, WI, USA), 0.25 pmol of each primer, 1.25U Taq polymerase (Promega, Madison, WI, USA), 10–100 ng of template DNA and water to a final volume of 25 µL. The PCR conditions used were an initial denaturation at 94 °C for 5 min, 35 cycles of denaturation (30 s at 94 °C), annealing (45 s at 55 °C), and extension (1.5 min at 72 °C).

Bacterial 16S rRNA genes were amplified by using the primers Eub27F and Eub1542R [24]. Each PCR reaction contained 1× PCR buffer with 3 mM MgCl2 (Promega), 400 µM dNTP mixture (Promega), 0.25 pmol of each primer, 1.25U Taq polymerase (Promega, Madison, WI, USA), 10–100 ng of template DNA and water to a final volume of 25 µL. Purified amplicons (~1500 bp) were cloned into a pGEMT-Easy vector (Promega, Madison, WI, USA) following the manufacture’s protocol. A total of 96 clones per sample were picked, and inserts were amplified with the M13F/R primers. After the screening, a Restriction Fragment Length Polymorphism (RFLP) assay was used to select different ribotypes of the clones. For each clone, 20 µL of the amplified insert (1600 bp) was restricted with an HhaI enzyme (Promega, Madison, WI, USA), and the product was separated by using 2% agarose gel electrophoresis [25]. Duplicates of each ribotype were reamplified and sequenced in Macrogen Inc. (Seoul, South Korea) by using the plasmid primers (M13F and M13R) on both sides of the insert. The obtained sequences were manually assembled and edited to resolve ambiguous positions by using ChromasPro software version 2.0.0 (Technelysium Pty Ltda, South Brisbane, Australia). Chimeras were identified by using Decipher [26] and removed prior to further analysis. The assembled sequences were submitted to the National Center for Biotechnology Information (NCBI) database under the accession number SUB6904016.

The closest relatives of the 16S rRNA sequences were determined by comparison with the SILVA database (SILVA database version 132 [27]) for taxonomic identification with default parameters (Operational Taxonomic Units (OTUs) at 98% similarity for genus level).

### 2.5. Bacterial 16S rRNA Gene Metabarcoding Analysis

Environmental samples were collected in November 2013 from P42, P53 (A, B and C) and P72. The samples were shaken vigorously and homogenized prior to DNA extraction. Total genomic DNA was extracted from 0.25 g of samples with a PowerBiofilm DNA Isolation kit (MoBio Laboratories Inc., Carlsbad, CA, USA) following the manufacturer’s instructions. DNA quality was evaluated by 1% *w/v* agarose gel electrophoresis and a NanoDrop 2000c spectrophotometer (Thermo Fisher Scientific, Waltham, MA, USA) at A260/280 nm (1.9–2.0).

The purified DNA from sediment and mats samples was amplified, and the V4 hypervariable region of the 16S rRNA were sequenced by using the bacterial primers 515F and 806R [28], which were optimized for the Illumina MiSeq platform. Sequencing was performed at the Research and Testing Laboratory (RTL, Lubbock, TX, USA) on a MiSeq (Illumina, San Diego, CA, USA) by using MiSeq Reagent kit (v2) with the longest read length set to 2 × 250 bp. The raw sequence data were submitted to the NCBI database under the accession number SUB6661100. 

From the total reads, PhiX and contaminant reads (e.g., Illumina adapters and barcodes) were removed. Raw sequences were filtered based on quality (Q > 20, *p* = 80) by FASTX-Toolkit, version 0.0.14 (http://hannonlab.cshl.edu/fastx_toolkit/). The assembling of reads was performed by using the Paired-End read mergeR (PEAR) assembly algorithm v 0.9.10 [29]. Paired end reads were selected after the quality filter was analyzed by using the open-source MOTHUR software v 1.39.5 [30], including alignment, clustering and dereplication. The sequences with incorrect length (<250 bp or >300 bp) or containing ambiguous bases (>0) and homopolymers (>8) were removed. The remaining sequences were processed by using the standard operating procedure of MOTHUR [31]. The taxonomical classification of each cluster was obtained by using SILVA database (SILVA database version 132) [27] with a confidence threshold of 98%. Unassigned sequences were removed by using the remove.lineage commands. The Ace, Chao1 and Simpson estimators of each sample were calculated by using the DOTUR (Defining Operational Taxonomic Units and Estimating Species Richness) function of MOTHUR. Rarefaction curves were obtained from all the samples with MOTHUR, and these curves were graphed with the R program.

The OTUs were analyzed by using Vegan package version 2.5-4 [32] in R version 3.4.4 (R Core Team, 2017). Community indexes were estimated for diversity (Rarefaction Species richness, Chao 1, Shannon, and Simpson index). Pielou’s evenness and sampling efforts were determined via the rarefaction curve. The coverage was calculated by Good’s method by using the formula [1 − (n/N)] × 100, where n is the number of singletons and N is the total number of reads analyzed [33].

A redundancy analysis (RDA) was performed to identify which environmental factors were driving the structure of microbial community for OTUs that were assigned at the phylum level by using rda function in vegan package in R.

### 2.6. Early Life Genera and Phylogenetic Analysis

An early life genera analysis was performed to determine the presence of deeply branching microbial groups in microorganisms with similar characteristics to the supposedly common ancestor for life in our data [7]. 

This analysis was developed by performing a search in our data for genera that had been described in the literature (Bergey’s Manual of Systematic of Archaea and Bacteria) as thermophile or thermotolerant, acidophile or acidotolerant, and anaerobe or facultative anaerobe (Table 2). Later, a phylogenetic analysis was performed by constructing a phylogenetic tree by using the 16S rRNA gene nucleotide sequences of the type strains or uncultured representatives of the genera that were selected for early life analysis. The nucleotide sequences were extracted from the Ribosomal Database Project (RDP) (RDP Release 11, Update 5, https://rdp.cme.msu.edu/index.jsp) and SILVA database (SILVA database version 132) [27] (Table S6). Moreover, four archaeal 16S rRNA gene nucleotide sequences (*Archaeoglobus profundus, Archaeoglobus veneficuspor, Thermoplasmata, acidophilum,* and *Thermoproteus tenax*) were used in this analysis. Nucleotide sequences were aligned by using MUSCLE (MEGA version 7.0.26). The distance matrix and phylogenetic tree were calculated according to the Tamura–Nei model [34], and maximum likelihood [35] algorithms with one hundred bootstraps were used to assign confidence levels to the nodes of the trees by using MEGA (version 7.0.26) software packages [36].

## 3. Results and Discussion

The Lirima hydrothermal system is composed of several springs in a physicochemical gradient of temperature (42 to 72 °C) and pH (5.2 to 7.8) (Table 1; Figure 1). The water corresponded to a hyposaline system (<0.89 psu) and low conductivity values (<1875 µS cm-1; Table 1). The temperature gradients were associated with shifts in chemical changes in the water. The high temperature spring (72 °C) was associated with higher bicarbonate, alkalinity, magnesium and fluoride levels compared with <53 °C springs (Table S1). These later springs were characterized by a slightly higher conductivity, as well as slightly higher sulfate, phosphate, and arsenic levels compared to the 72 °C spring. Furthermore, the springs did not present a significant change in the physicochemical parameters through time, maintaining a stability in their temperature, pH, salinity and conductivity in different seasons and even different years of sampling (Table 1). Previous reports have supported our results, showing similar gradients of temperature and pH and a deep circulation from a geothermal reservoir, with a low mixing degree in the Lirima hydrothermal system [14,37]; however, we registered a more acidic pH (5.2) in the hottest spring (P72). Moreover, high concentrations of CO_2_ (596.4 µM) and CH_4_ (38,412 nM) in P72 compared to P42 (CO_2_, 156.9 µM; CH_4_, 6,123 nM; Table S1) and P53 (CO_2_, 124.8 µM; CH_4_, 3,807 nM; Table S1) were measured.

The analyses of the archaeal and bacterial diversity of the microbial mats in the Lirima hot springs that were performed in this study started on April 2011 by using 16S rRNA gene clone libraries. In November 2013, a more sensitive and high-throughput screening was performed to further analyze the bacterial diversity of the microbial mats and sediment samples of the Lirima hot springs by using 16S rRNA gene metabarcoding. We would like to emphasize that the two different techniques used for microbial community analyses are not directly comparable; rather, they complemented each other, as each provided strengths and weaknesses to microbial diversity analyses. 

**Figure 2:**
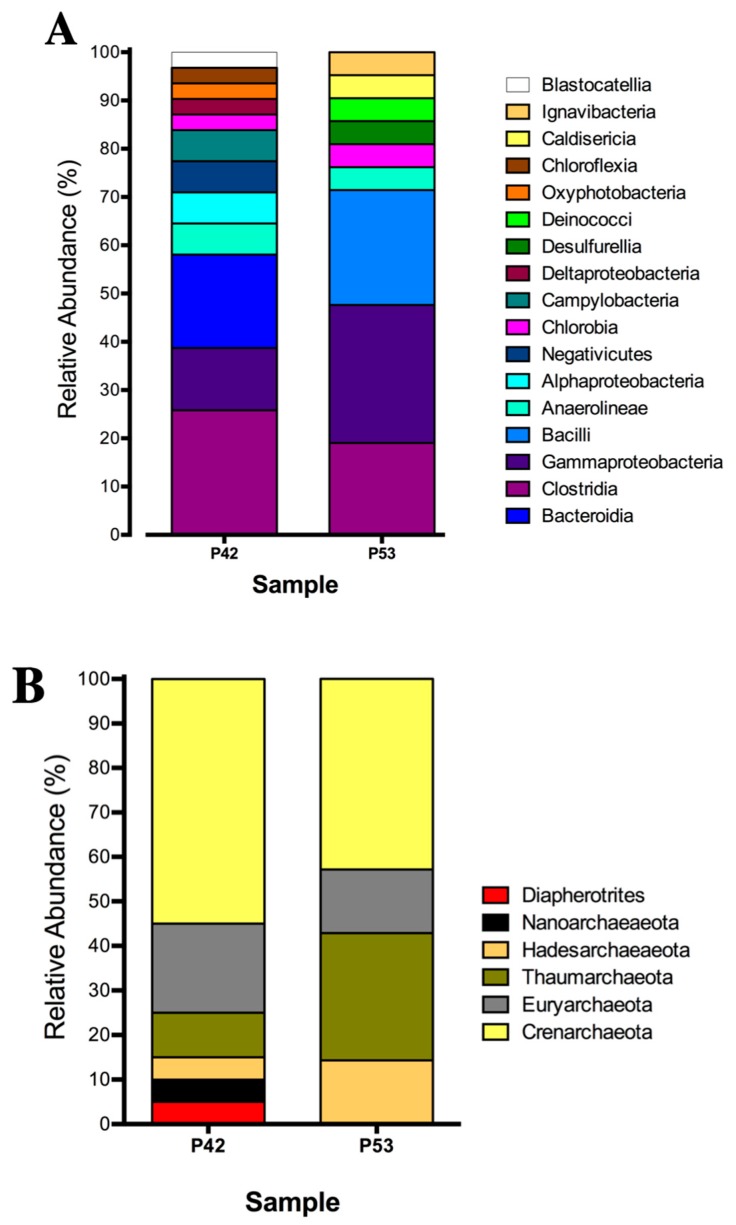
Relative abundance of the 16S rRNA gene clonal analysis that was classified at the phylum level of the microbial community structure from the hot springs at the Lirima hydrothermal system. (**A**) Bacterial diversity of the P42 and P53 springs. (**B**) Archaeal diversity of the P42 and P53 springs.

The bacterial community compositions that was determined by 16S rRNA gene clone libraries analyses of the P42 and P53 springs were mainly represented by anaerobic, microaerophilic and aerobic thermophiles, with some of these being associated with sulfate reducers or sulfide oxidizing metabolisms (Table S2A). The P42 spring was composed mainly by OTUs that were classified as Clostridia (26%), Bacteroidia (19%) and Gammaproteobacteria (13%) (Figure 2A; Table S2A). Moreover, the P53 spring was highly represented by Gammaproteobacteria (29%), Bacilli (24%) and Clostridia (19%) (Figure 2A; Table S2A). On the other hand, the archaeal community compositions that was determined by 16S rRNA gene clone libraries analyses of the P42 and P53 springs could only be assigned to higher taxonomic levels (class, order) (Figure 2B; Table S2B) and were also associated with anaerobic and thermophilic taxa belonging to Crenarchaeota, Diapherotrites, Nanoarchaeota, Hadesarchaeota, Euryarchaeota; as well as an aerobic ammonia oxidizer from Thaumarchaeota. The core groups (detected in both springs) presented changes in their contributions that were potentially associated with the different physical and chemical conditions. For example, the Crenarchaeota phylum decreased its contribution in our libraries from 55% to 43% (P42 vs. P53), and a single class (Thermoplasmata) from the Euryarchaeota phylum decreased its contribution in our libraries from 20% to 14% (Figure 2B; Table S2B), whereas specific archaea, such as Diapherotrites and Nanoarchaeota, were detected only at the P42 spring, (Figure 2B; Table S2B).

Both Euryarchaeota and Crenarchaeota are widely found in hot springs all over the world [38,39,40]. Furthermore, Nanoarchaeota comprise a diverse group of archaea that are capable of inhabiting a broad spectrum of temperatures and geochemical environments, and this phylum has been found to be widespread in terrestrial hot springs including a hydrothermal sediment sample from the Géiseres del Tatio volcanic region in Chile at the Atacama Desert [41,42].

**Figure 3:**
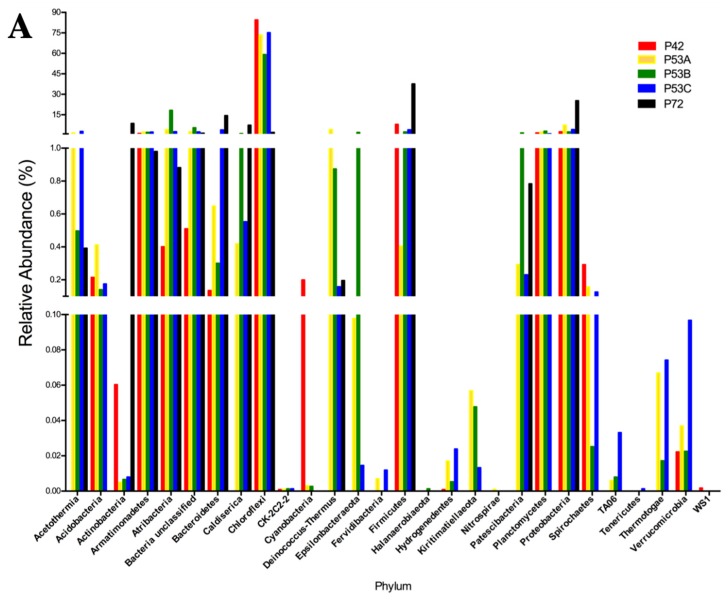

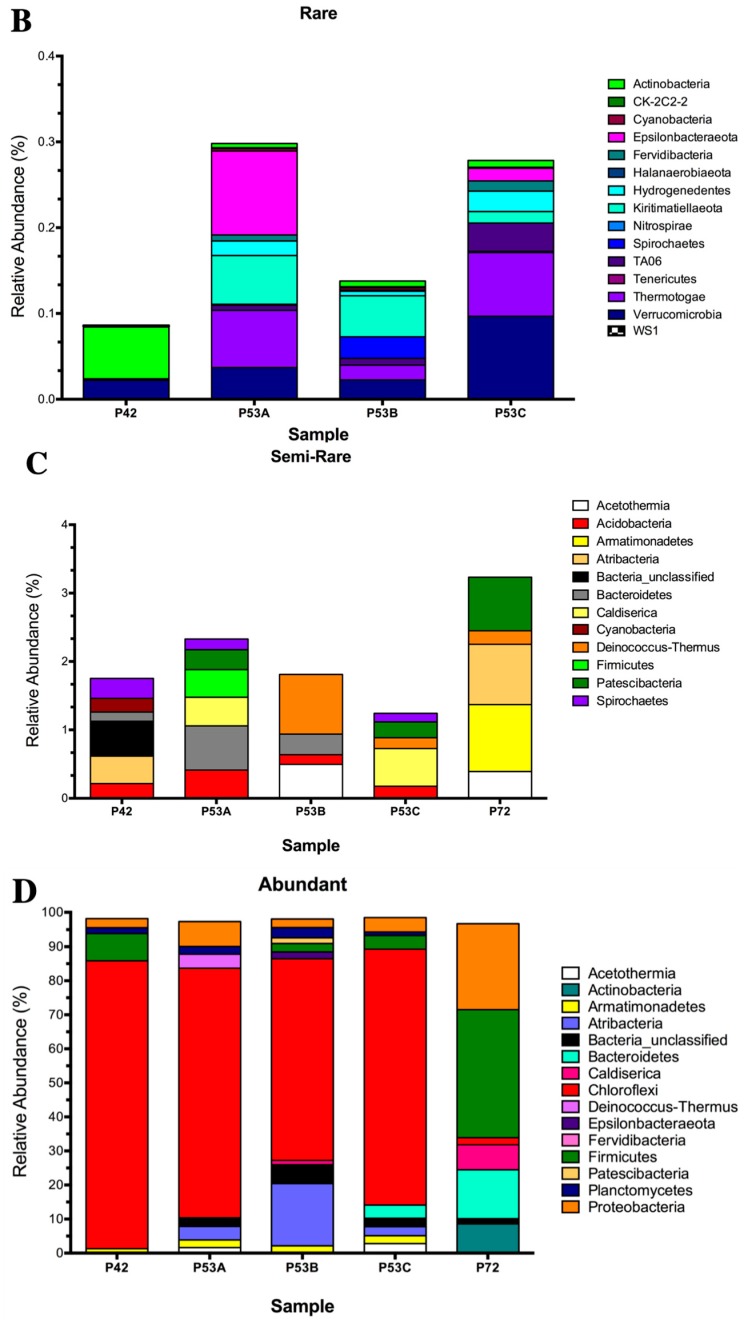
Relative abundance of the 16S rRNA gene metabarcoding analysis that was classified at the phylum level of the microbial community structure from the hot springs at the Lirima hydrothermal system. (**A**) Bacterial diversity of the P42, P53A, P53B, P53C and P72 springs. (**B**) Rare bacterial diversity of the P42, P53A, P53B and P53C springs. (**C**) Semi-rare bacterial diversity of the P42, P53A, P53B, P53C and P72 springs. (**D**) Abundant bacterial diversity of the P42, P53A, P53B, P53C and P72 springs.

The sequencing results of the bacterial 16S rRNA metabarcoding analysis from three springs (P42, P53A, P53B, P53C, and P72) varied from 1021 (P72) to 112,667 (P42) reads (Table S3). A total of 70 to 429 OTUs were taxonomically assigned to each spring, but only 21 OTUs were represented by >1000 reads (Table S3). The rarified species richness varied between 32.8 and 70 (Table S3; Figure S1) and the estimated richness based on Chao1 varied between 73 and 867 (Table S3). The Simpson’s index varied from 0.463 to 0.926. Good’s estimator showed a coverage of >99% for each site (Table S3), and the rarefaction analysis indicated that the curve did not reach an asymptote, particularly for P72 (Figure S2). The P42 and P53 (sediment A, B, C) springs showed the highest bacterial diversity compared with P72 at the phyla level (16–24 compared with 12; Figure 3A versus 3D). Moreover, the coolest spring sediments and mats (≤53 °C) presented a higher diversity of rare and low abundant phyla, including phyla like Actinobacteria and Firmicutes, which showed an increased contribution in the hottest spring (Figure 3B). Conversely, P72 did not present a rare biosphere, and its bacterial diversity was mainly represented by a few abundant phyla (12, P72; Figure 3D) and a lower representation of the semi-rare biosphere (Figure 3C). Li and colleagues [43], by using a culture-independent approach, determined that in the Tengchong geothermal field (China), microbial communities at higher temperatures (74.6–90.8 °C) had substantially lower diversity than those growing at a low temperature, showing only a few genera and a lower number of cells. Generally, it has been reported that thermal environments exhibit a lower microbial diversity, which is probably related to the unique development of specific adaptation strategies by these microorganisms to thrive under harsh conditions (see below). Furthermore, P72 had only 1021 sequences, and this could have limited the assignation of a higher number of OTUs. In some types of microorganisms, DNA extraction may be more difficult, as some bacteria and archaea are resistant to cell disruption [44], especially in samples from high temperatures and more acidic springs such as P72.

Potentially, the core bacterial communities at the Lirima hydrothermal system were Chloroflexi in springs with lower temperatures (84%, P42; 73%, P53A; 59%, P53B; and 75%, P53C), followed by Firmicutes, which reached up to 38% in the hottest spring (Figure 3A; Table S4). Other phyla, such as, Acetothermia, Atribacteria and Epsilonbacteraeota, were also prevalent in the sediment and mats of P53, and only low frequency groups, such as Fervidibacteria, Kiritimatiellaeota, TA06, and Thermotogae, were detected at this site (Figure 3A; Table S4). OTUs that were assigned as unclassified bacteria were found in all the springs (between 0.5% and 5.5%; Table S4), which could correspond to a high proportion of novel taxa in this complex ecosystem.

Thermophilic phototrophy could be a significant process in hydrothermal ecosystems when considering the presence of species such as Chloroflexi as a core microbial group, especially in springs with lower temperatures (42 and 53 °C) where it has shown a high abundance (Figure 3A). Photoautotrophic carbon fixation might be one of the most important biosynthetic processes in these oligotrophic habitats, especially in springs with a high availability of CO_2_, such as P72 [45,46,47]. Moreover, the presence of phototrophic microorganisms helps to increase the level of dissolved oxygen in hot waters, which is needed for the growth of aerobic or microaerophilic microorganisms in these communities [48]. The low abundance of sequences that were assigned to Chloroflexi (2%) in the P72 spring could be explained by the temperature (72 °C) and pH (5.2) of the spring, since it has been proposed that the upper temperature limit for phototrophic metabolism in neutral-to-alkaline springs is 73 °C, and it decreases with decreasing pH [49].

Phototrophic sulfur bacteria oxidize various inorganic sulfur compounds, such as sulfide, elemental sulfur, thiosulfate, ferrous iron and hydrogen, for use as electron donors in anoxygenic photosynthetic growth [50]. Therefore, sulfur bacteria are responsible for energy production, which helps to maintain the community structure [51]. Bacteria oxidize sulfur compounds for autotrophic CO_2_ fixation in reducing habitats (e.g., hydrothermal systems) and play a critical role in primary production and sulfur transformation [52,53]. The taxa that were retrieved by metabarcoding analysis (Table S3) showed sulfate or sulfite reducing bacteria, as well as anaerobic, microaerophilic and thermotolerant or thermophilic organisms. Sulfur bacteria belonging mostly to the Deltaproteobacteria class were found in the springs and included *Desulfatirhabdium, Desulfobulbus, Desulfomicrobium, Desulfomonile, Desulfosoma, Desulfovermiculus, Desulfurella, Desulfurispora, Dissulfurimicrobium* and *Thermodesulfitimonas* (Table S3).

Sequences that were assigned as uncultured were associated with novel phyla, such as the candidate phylum Armatimonadetes (OP10), candidate phylum Atribacteria (OP9/JS1), candidate phylum Acetothermia (OP1), and candidate phylum Caldiserica (OP5), all of which were detected with higher relative abundance in the P53 spring (Figure 3A). These candidate phyla of heterotrophic anaerobes have been also previously detected in geothermal springs in Yellowstone National Park in USA [54], Mae Fang hot spring in Thailand [55] and Tengchong thermal springs in China [56], which suggests a strong adaptation to hyperthermophilic environments. Different cultivation-independent genomic approaches have suggested that thermophilic heterotrophic anaerobes are potentially related to the primary fermentation of carbohydrates or the secondary fermentation of organic acids that are present in thermal environments [57].

### 3.1. Adaptation to Environmental Factors

**Figure 4:**
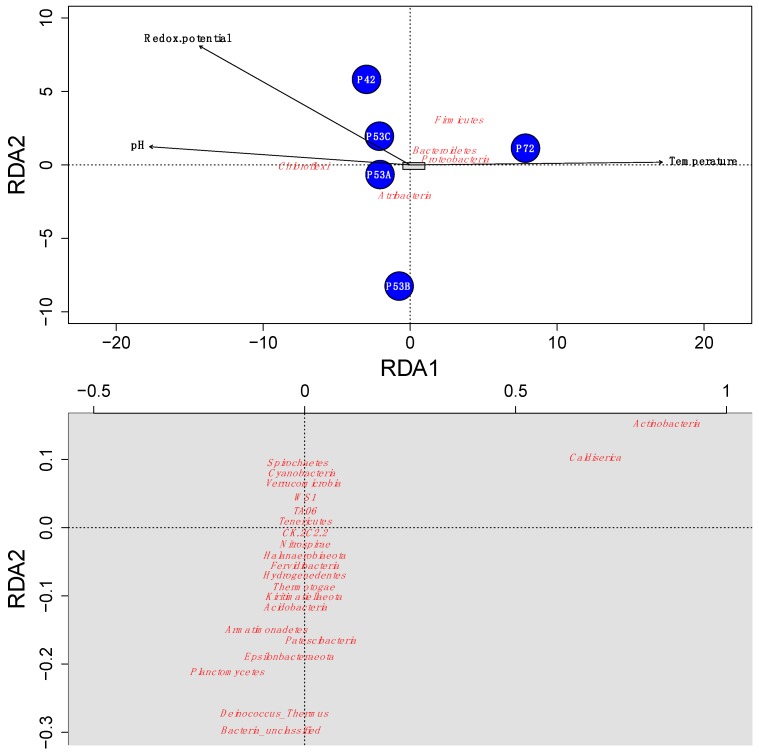
Redundancy analysis (RDA) ordination plot that illustrates the relations between the Lirima hot springs’ microbial community composition (red) and the environmental variables that explain most of the variance (black) in each of the springs (blue circles). The grey rectangle in the first plot (above) is represented in the second plot (below) with the ordination of the species with less abundance. The ordination of the species in the second plot was rearranged when plotted to accomplish a better resolution.

The microbial community structure varied at the different springs of the Lirima hydrothermal system. A redundancy analysis indicated that the temperature, redox potential, and pH explained ~96% of the variance that was determined in the springs and revealed that changes in temperature greatly influenced the microbial community in the mats and sediments between 53 and 72 °C (Figure 4). This factor could select for and drive changes within these high-altitude thermophilic microbial communities, especially for Atribacteria, Chloroflexi, and Firmicutes, which were the most abundant phyla in the springs (Figure 4; Table S5). Our results support previous studies that have shown that significant differences in microbial communities exist among hot springs with different ranges of physicochemical parameters and discrete geographic locations [58,59,60]. Furthermore, Cole et al. [61] proposed that temperature can control the richness and composition of sediment communities at all taxonomic levels, possibly with the factors that covary with temperature in geothermal systems, due to processes such as degassing, mineral precipitation, evaporation, autotrophy and oxidation [62,63]. Therefore, adaptation mechanisms to stress-inducing environmental factors are important in microbial communities of extreme environments and could potentially explain their success in these types of ecosystems, as well as their evolution over time [18,19,64,65].

A high representation of Firmicutes in the hottest spring (P72) in the metabarcoding analysis and in the springs with lower temperatures (P42 and P53) shown by clonal analysis could be explained by a myriad of survival strategies to resist adverse conditions that have been previously described in this taxa (e.g., sporulation, metabolism adaptation/re-programming) and which represent one of the most ubiquitous microbial groups [66]. Fillippidou et al. [67] determined that there was a higher contribution of endospore-forming Firmicutes (EFF) at sites with multiple limiting factors such as the geothermal springs in Lirima. Recently, Filippidou et al [17] reported that different simultaneous stresses (e.g., heat shock stress, UV radiation and starvation) induce the diversification of *Serratia ureilytica*, a traditionally non-spore forming bacteria, into two populations, and they suggested that the occurrence of cell resting states could represent a successful strategy to cope with environmental polystresses.

**Figure 5:**
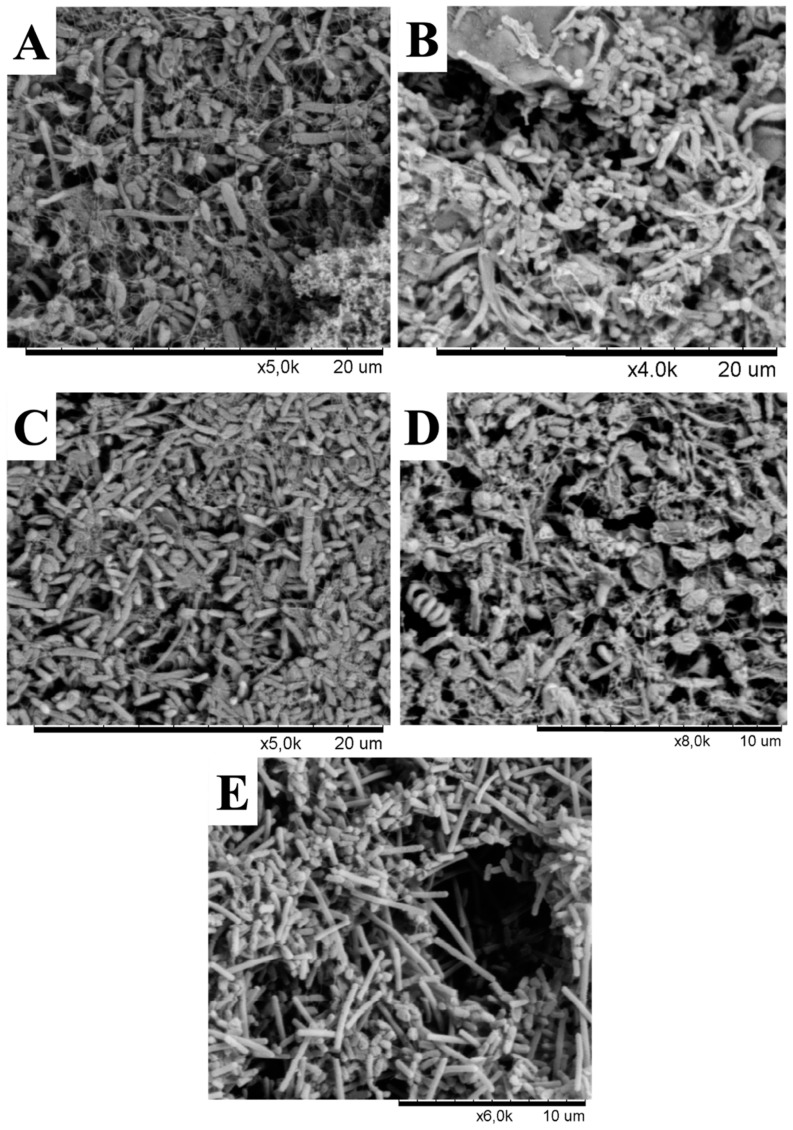
Scanning electron microscopy micrographs from enrichment cultures of hot springs. (**A**) P42, (**B**), P53A, (**C**), P53B, (**D**) P53C, and (**E**) P72.

In addition to changes in phylotypes, our results demonstrated that microbial communities in the Lirima hydrothermal system also show an overall high difference in their morphotypes that were found in all springs (Figure 5). It should be noted that the aim of the scanning electron microscopy (SEM) images was not to estimate or quantify the diversity of the original natural communities but to visualize the presence of microorganisms in the samples and to distinguish different morphotypes between springs. The images showed that a rod-shaped morphology was more elongated and filamentous in the hottest spring (P72; Figure 5E), compared with springs with lower temperatures (P42, P53A, P53B and P53C; Figure 5A–D, respectively). Different morphotypes and cell sizes of microorganisms have often been described in hot springs and as being related to changes in microbial community structure according to their adaptation to environmental conditions of the spring water (temperature, pH and sulfur concentration) [68]. Furthermore, these differences could also be explained by morphological variation within phylotypes according to growth conditions, as described in hyperthermophilic microorganisms, such as *Thermocrinis ruber*, a pink- filament-forming bacterium that was isolated from Yellowstone National Park and which exhibits some degree of phenotypic plasticity [69].

The survival abilities of these communities could be increased through strategies such as mutualistic or commensalist symbiotic relationships [70] and lateral gene transfer, which would allow the acquisition of new functions to adapt to their environment [71]. However, further studies must be performed to define their environmental resistome [65].

### 3.2. Lirima Hot Spring: A Doorway for Early Life

Geologic evidence indicates that the first traces of life on Earth originated 3.8–4.2 Ga Year ago [5,72], and that primitive Earth had higher temperatures compared to modern Earth; therefore, one of the theories about the origin of life suggests that it could had occurred in hot environments like modern thermal systems [7]. Furthermore, hydrothermal systems appear to have played a fundamental role in the early evolution of Earth, as they linked the global lithospheric, hydrologic and atmospheric elemental cycles [73]. Over geologic time, volatile chemicals released by hydrothermal systems have significantly contributed to the evolution of the oceans and the atmosphere [74]. Hydrothermal systems contain important biogeological information that suggest these environments have played an important role in the history of the biosphere [74].

**Figure 6:**
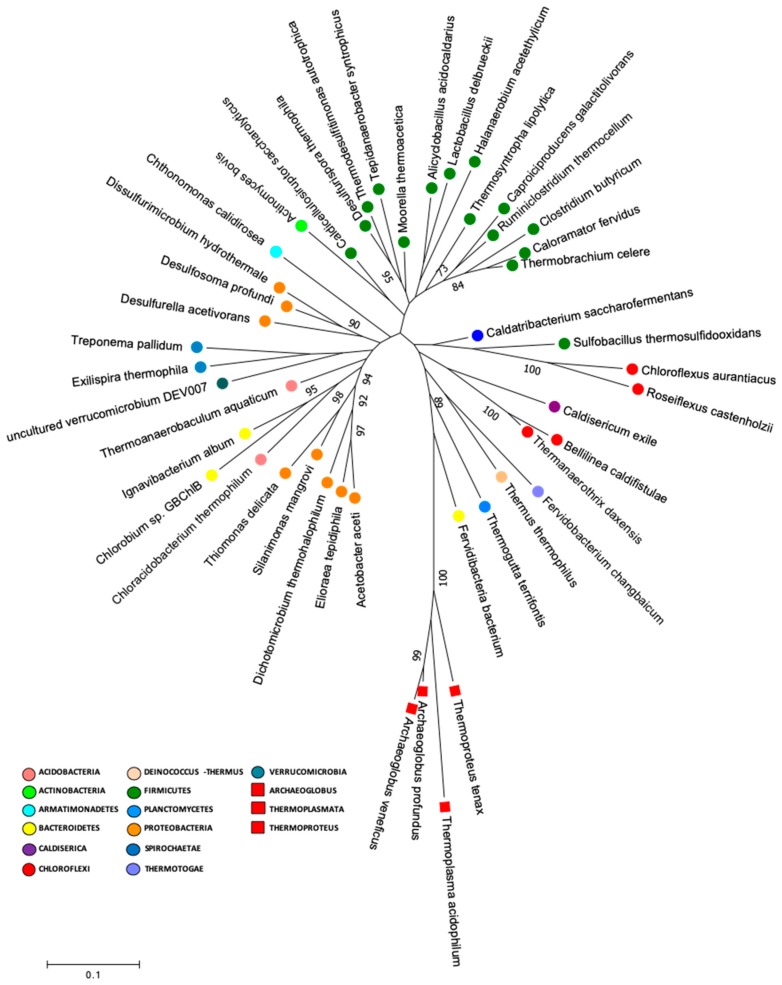
Molecular phylogenetic analysis of early life genera. The evolutionary history was inferred by using maximum likelihood method based on the Tamura–Nei model with one hundred bootstraps to assign confidence levels to the nodes of the trees, (MEGA version 7.0.26).

In the present study, we performed a search in our data for species that had similar characteristics to the supposedly common ancestor for life, which included thermophilic or thermotolerant, acidophilic or acidotolerant, anaerobic or facultative anaerobic metabolisms. Our results showed that the Lirima springs presented 41 genera that were described as anaerobic thermophilic, thermotolerant, acidophilic and or acidotolerant, that were distributed between 14 phyla, and that were highly represented by Firmicutes (Table 2). Furthermore, we performed a phylogenetic analysis to determine the presence of deeply branching microbial groups in our data by constructing a phylogenetic tree by using 16S rRNA gene sequences of the type strains of these 41 genera (Figure 6; Table S6). The phylogenetic tree showed a cluster of sequences that were closely grouped to two archaea (*Archaeoglobus veneficuspor* and *Archaeoglobus profundus*) that are commonly located in the deepest branch of the tree of life [7]. In this group, there were mainly Gram-negative bacterial genera, among which we found *Chloroflexus, Roseiflexus, Caldisericum, Bellilinea, Thermanaerothrix, Fervidobacterium, Thermus, Thermogutta,* and *Fervidibacteria* (Figure 4). These genera have been described with fermentative, chemoorganotrophic, autotrophic and mixotrophic metabolisms. Furthermore, Weiss et al. [7] suggested an autotrophic origin of life. Among phototrophs, the genus *Chloroflexus* and its filamentous relatives, such as *Roseiflexus* and *Thermanaerothrix*, have been located in the deepest phylum on the bacterial branch of the tree of life [75].

The phylogenetic information that is encoded in the genomes of modern thermophilic species appears to provide important clues about early biosphere evolution and the processes that shaped its history [2,74]. The deepest branches, and therefore those nearest to the common ancestor of all life (which was probably a chemolithoautotrophic, thermophilic and anaerobic organism), are represented by hyperthermophilic chemosynthetic microorganisms that use hydrogen and sulfur in their metabolism [1,6,7], suggesting that these environments may have been a “cradle” for the early biosphere and supporting the ideology that they are the “Doorway to Early Biosphere Evolution” [74]. Therefore, the high altitude hydrothermal environments of the Chilean Altiplano (~4000 m a.s.l.) are unique ecosystems that are dominated by microbial life whose hyper extreme conditions and low anthropogenic perturbations offer a natural laboratory to better understand the early processes that underlie the life forms we know today. Recently, Hug et al. [76], in “a new view of the tree of life” (including bacteria, archaea and eukaryotes), showed that the inclusion of uncultivated representatives (either rRNA sequences or functional genes) allows for a better taxonomic resolution in a phylogenetic analysis. Nonetheless, several taxa remain unresolved. Therefore, it is expected that using phylogenetic approaches that are based on metagenomic data and proteomics will give the most robust image of LUCA’s fingerprint found in the genes of modern organisms, especially from remote and understudied thermal ecosystems such as the Lirima hydrothermal system. Therefore, this will certainly be considered in future studies of this site. 

## 4. Conclusions

In this study, microbial community diversity in the mats (P42 and P53A) and sediments (P53B, P53C and P72) of the Lirima hydrothermal system was investigated by using a metabarcoding analysis of the V4 hypervariable region of the 16S rRNA gene and 16S rRNA clonal analysis. The results revealed that the hot springs were characterized by an overall predominance of phototrophic bacteria, including Chlorobi, Chloroflexi and Cyanobacteria in springs with lower temperatures (P42 and P53) and a predominance of Firmicutes in warmer springs (P72). Microbial communities in Lirima are influenced by site-specific environmental factors, mainly temperature, which would select and probably drive changes in their composition, despite the geographical proximity between hot springs. Furthermore, our results also showed a high representation of metabolically versatile prokaryotes, such as Chloroflexi and Deltaproteobacteria, which are able to respond to changes in environmental factors.

Early Earth probably had a chemically reducing atmosphere, and primary life could have happened in thermal environments (e.g., hydrothermal systems); therefore, the study of modern hydrothermal ecosystems could provide an opportunity to have a better comprehension about the evolution of earliest life in the primitive atmosphere. Our study identified sequences that were associated with prokaryotes that have an anaerobic, thermophilic, thermotolerant, acidophilic and acidotolerant metabolism, which has been related to the metabolism that the common ancestor of life (LUCA) probably possessed.

Inland geothermal systems at high altitude have not been studied in detail as analogs of primitive Earth. Thus, the Lirima hydrothermal system represents a key system for the analysis of the microbial genomes of thermophilic and hyperthermophilic species from deep branches of the tree of life that could help reconstruct the evolutionary history of microbial communities in hydrothermal systems for Chile and the world. In this regard, the study and preservation of the microbial communities of these fragile ecosystems could expose the deep secret of primitive life and give evidence about the what kind of life we could expect to find on exoplanets.

## Figures and Tables

**Table 1:** Physicochemical parameters of the Lirima hot springs.

Physicochemical Parameters	Sampling Year	P42	P53A	P53B	P53C	P72
Temperature (°C)	2011	45	51	51	51	ND
	2013	42	48	48	53	72
pH	2011	7.92	7.6	7.6	7.6	ND
	2013	7.8	7.2	7.2	7.2	5.2
Conductivity (µS/cm)	2011	2520	2100	2100	2100	ND
	2013	1837	1875	1875	1875	1848
Salinity (psu)	2011	1	1	1	1	ND
	2013	0.89	0.71	0.71	0.71	0.86
Redox potential (mV)	2013’	−152.4	−246.7	−246.7	−246.7	−316.9
Type of Soil		Mat	Mat	Sediment	Sediment	Sediment
Altitude (m a.s.l.)		4016	4016	4016	4016	4,016
GPS coordinates		19°51′7.75″S; 68°54′24.20″W	19°51′7.75″S; 68°54′24.20″W	19°51′7.75″S; 68°54′24.20″W	19°51′7.75″S; 68°54′24.20″W	19°51′5.69″S; 68°54′23.57″W

**Table 2:** Genera described as aerobic, anaerobic thermophilic, thermotolerant, acidophilic and or acidotolerant of the Lirima hot springs.

Characteristics^1^	Genus	Phylum	Metabarcoding^2^					Clones^2^	
			P42	P53A	P53B	P53C	P72	P42	P53
◯■❖	*Chloracidobacterium*	Acidobacteria							
◯■♦	*Thermoanaerobaculum*	Acidobacteria							
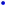 ☐	Actinomyces	Actinobacteria							
◯■	Chthonomonadales	Armatimonadetes							
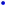 ■	Candidatus *Caldatribacterium*	Atribacteria							
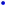 ☑	Fervidibacteria	Bacteroidetes							
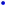 ■	GBChlB	Bacteroidetes							
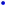 ■	*Ignavibacterium*	Bacteroidetes							
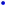 ■	*Caldisericum*	Caldiserica							
◯☐	*Bellilinea*	Chloroflexi							
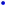 ■	*Chloroflexus*	Chloroflexi							
◯■	*Roseiflexus*	Chloroflexi							
◯■	*Thermanaerothrix*	Chloroflexi							
◯■	*Thermus*	Deinococcus–Thermus							
◯♦■ 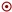	*Alicyclobacillus*	Firmicutes							
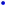 ■	*Caldicellulosiruptor*	Firmicutes							
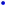 ■ 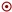	*Caloramator*	Firmicutes							
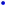 ☐	*Caproiciproducens*	Firmicutes							
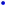 ☐	*Clostridium sensu stricto*	Firmicutes							
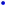 ■ 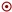	*Desulfurispora*	Firmicutes							
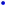 ☐	Halanaerobium	Firmicutes							
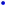 ❖	*Lactobacillus*	Firmicutes							
◯■ 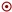	*Moorella*	Firmicutes							
◯■	*Ruminiclostridium*	Firmicutes							
◯☐♦ 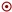	*Sulfobacillus*	Firmicutes							
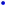 ■ 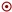	*Tepidanaerobacter*	Firmicutes							
◯■	*Thermobrachium*	Firmicutes							
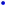 ■	Thermodesulfitimonas	Firmicutes							
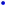 ■	*Thermosyntropha*	Firmicutes							
◯■	*Thermogutta*	Planctomycetes							
◯❖■	*Acetobacter*	Proteobacteria							
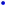 ■	*Desulfosoma*	Proteobacteria							
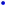 ■ 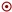	*Desulfurella*	Proteobacteria							
◯■	*Dichotomicrobium*	Proteobacteria							
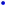 ■	*Dissulfurimicrobium*	Proteobacteria							
◯■	*Elioraea*	Proteobacteria							
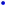 ■	*Silanimonas*	Proteobacteria							
◯ ❖	*Thiomonas*	Proteobacteria							
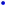 ■	*Exilispira*	Spirochaetae							
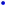 ■	Treponema	Spirochaetae							
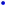 ■	*Fervidobacterium*	Thermotogae							
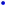 ■	DEV007	Verrucomicrobia							

^1^ Symbols representing each characteristic; ^2^ Relative abundance color code; 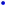 Anaerobic; ◯ Aerobic; ◯ Facultative anaerobic; ■ Thermophilic; ☐ Thermotolerant; ☑ Hyperthermophilic; ♦ Acidophilic; ❖ Acidotolerant; and 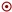 Spore former; Highlight in blue: <0.1%; Highlight in yellow: 0.1–1%; Highlight in bright green: >0.1%.

## Supplementary Materials

Supplementary materials can be found at https://www.mdpi.com/2076-2607/8/2/208/s1.
