# Supplementary figures and images for: Aquatic Thermal Reservoirs of Microbial Life in a Remote and Extreme High Andean Hydrothermal System
Source: Microorganisms. 2020 Feb 3;8(2):208. doi: 10.3390/microorganisms8020208 (PMC7074759; doi:10.3390/microorganisms8020208)

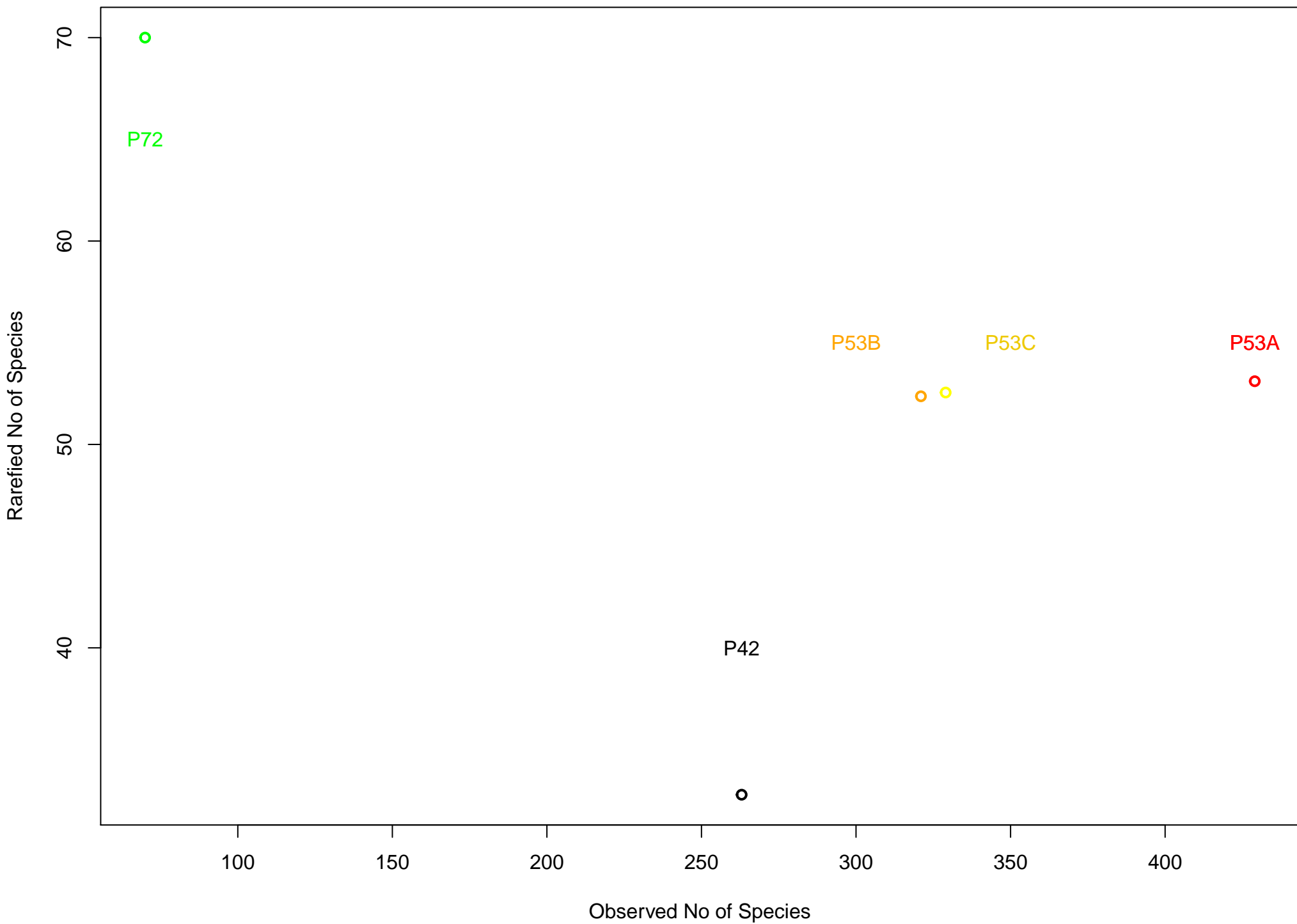

Supplement: Supplementary file 1 [file microorganisms-08-00208-s001.zip › Supplementary Figure 1.pdf]

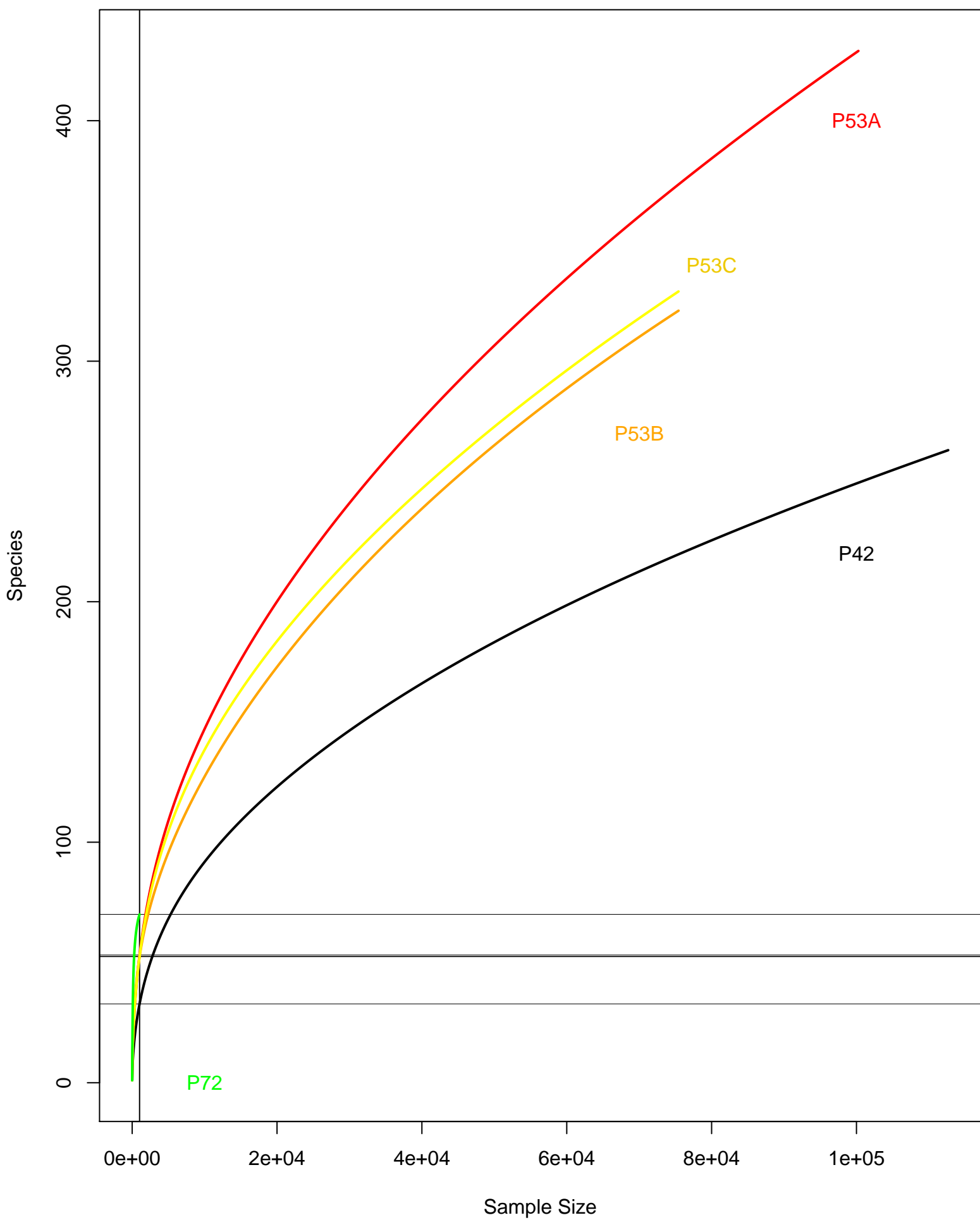

Supplement: Supplementary file 1 [file microorganisms-08-00208-s001.zip › Supplementary Figure 2.pdf]
